# miR-sc8 Inhibits Schwann Cell Proliferation and Migration by Targeting Egfr

**DOI:** 10.1371/journal.pone.0145185

**Published:** 2015-12-18

**Authors:** Yun Gu, Chu Chen, Sheng Yi, Shanshan Wang, Leilei Gong, Jie Liu, Xiaosong Gu, Qing Zhao, Shiying Li

**Affiliations:** 1 Jiangsu Key Laboratory of Neuroregeneration, Co-innovation Center of Neuroregeneration, Nantong University, Nantong, Jiangsu, P. R. China; 2 Department of Obstetrics and Gynecology, Affiliated Hospital of Nantong University, Nantong, Jiangsu, P. R. China; 3 Key Laboratory of the People’s Liberation Army, Institute of Orthopaedics, Chinese PLA General Hospital, Beijing, P. R. China; University of Saarland Medical School, GERMANY

## Abstract

MicroRNAs (miRNAs) negatively regulate the expression of target genes at the post-transcriptional level in diverse biological processes. We have previously identified a group of novel miRNAs in proximal nerve following rat sciatic nerve transection by Solexa sequencing. In this study, the biological function and action mode of miR-sc8, one of the above identified miRNAs, were investigated. An increased expression of miR-sc8 inhibited cell proliferation and migration of Schwann cells (SCs), and inversely, silencing of the miR-sc8 expression promoted cell proliferation and migration of SCs. The epidermal growth factor receptor (Egfr) was identified as the target gene of miR-sc8, which exerted negative regulation of Egfr by translational suppression. The temporal change profile of the miR-sc8 expression was negatively correlated with that of the Egfr expression in proximal nerve following sciatic nerve transection. Moreover, Knockdown of Egfr attenuated the promoting effects of miR-sc8 inhibitor on SC proliferation and migration. Overall, our data indicate that miR-sc8 affects phenotype modulation of SCs by targeting Egfr, providing further insights into the regulatory role of miRNAs in peripheral nerve regeneration.

## Introduction

MicroRNAs (miRNAs) refer to a class of small non-coding RNAs with a length of about 22 nucleotides [1]. They can negatively regulate the expression of a variety of genes in diverse physiological and pathological processes by interacting with the 3’-untranslated regions (3’-UTRs) of their corresponding targets [2–4]. Recently, the temporal expression profiles of miRNAs after peripheral nerve injury have been determined in different animal models. Mounting evidence indicates that these differentiated expressed miRNAs significantly affect the biological behaviors of neurons and Schwann cells (SCs), including survival maintenance, axonal regrowth and phenotype modulation [5–9].

As the main glial cells in the peripheral nervous system (PNS), SCs play key roles in the development and regeneration of PNS due to their interactions with growing axons [10]. Based on this, phenotype modulation of SCs, especially promotion of SC proliferation and migration, has been extensively investigated for improving peripheral nerve regeneration. Very interestingly, emerging data have indicated that miRNA regulation of gene expression in SCs is able to affect SC proliferation and migration, axon myelination, and axonal integrity, suggesting that some miRNAs could be exploited as therapeutic targets for peripheral nerve injury [5, 8, 11–13].

Given the importance of miRNA in peripheral nerve regeneration, we are consistently interested in the expression profiles and regulatory roles of miRNAs in peripheral nerve injury and regeneration. To identify novel miRNAs that might be involved in nerve regeneration, a rat model of sciatic nerve transection is extensively used. Solexa sequencing has helped us to identify that a number of novel miRNAs are differentially expressed in the injured nerve or in dorsal root ganglia after sciatic nerve injury in rats [14, 15]. Briefly, Solexa sequencing was performed by the Beijing Genomics Institute (Shenzhen, China). The small RNAs were ligated with a pair of Solexa adaptors to their 5’ and 3’ ends, and ligation products were treated for sequencing. For the relevant technical details please refer to our previous publications [14, 15]. Among these identified miRNAs, miR-sc8 was selected as the object of this study because the biological functions of miR-sc8 and its action mode could be clearly elucidated. We found that miR-sc8 inhibited the proliferation and migration of SCs by directly targeting the epidermal growth factor receptor (Egfr).

## Materials and Methods

### Primary culture of SCs and oligonucleotide transfection

Neonatal 1-day-old Sprague-Dawley (SD) rats were obtained from the Experimental Animal Center at Nantong University, China. The SCs were isolated from the sciatic nerve of rats and purified by removing fibroblasts, followed by cell characterization as previously described [13]. Primary culture of SCs was maintained in Dulbecco’s modified Eagle’s medium (DMEM) containing 10% fetal bovine serum (FBS) in a humidified 5% CO_2_ incubator at 37°C.

Primary SCs were transfected with miR-sc8 mimic, and miR-sc8 inhibitor, non-targeting negative control, or Egfr siRNA (Ribobio, Guangzhou, China), respectively, using Lipofectamine RNAiMAX transfection reagent (Invitrogen, Carlsbad, CA) according to manufacturer’s instructions.

### Cell proliferation assay

The proliferation rate of SCs was measured using the Cell-Light^TM^ EdU DNA Cell Proliferation Kit (Ribobio) as previously described [13]. Briefly, primary SCs were resuspended in fresh pre-warmed (37°C) medium, counted, and seeded at a density of 2×10^5^ cells/ml onto 0.01% poly-L-lysine-coated 96-well plates. After 5-ethynyl-2’-deoxyuridine (EdU) was applied to cell culture, and cells were allowed to incubate for additional 24 h, the cells were fixed with 4% formaldehyde in phosphate buffered saline (PBS) for 30 min. Hoechst 33342 nucleus staining was also applied for the cells. The cell proliferation of primary SCs was analyzed from the images of randomly selected fields obtained on a DMR fluorescence microscope (Leica Microsystems, Bensheim, Germany). Analyses were performed three times using triplicate wells.

### Cell migration assay

The cell migration of primary SCs was assessed by transwell-based assay as described previously [13]. The 6.5-mm transwell chambers with 8 mm pores (Costar, Cambridge, MA) were used, and the bottom surface of each membrane was coated with 10 μg/ml fibronectin. Primary SCs (3×10^5^ cells/ml, 100 μl) were placed onto the upper chamber of each transwell, the cells were allowed to incubate at 37°C in 5% CO_2_, and 600 μl complete medium was added to the lower chambers. SCs on the upper surface of the membranes were removed using a cotton swab and SCs on the lower surface were stained with 0.1% crystal violet for 15 min at room temperature, imaged and randomly counted using a DMR inverted microscope (Leica Microsystems). Assays were performed three times using triplicate wells.

### Luciferase assay

The 3’-UTR sequence of Egfr, Cx3cl1, Cdk5r1, Rac2, or Plat was amplified from the genomic DNA and subcloned into the region directly downstream of the stop codon in the luciferase gene in the luciferase reporter vector. Overlap PCR was used to construct 3’-UTR mutant reporter plasmid. HEK 293T cells were cultured in 24-well plates and transfected with a mixture of p-Luc-UTR, miRNA mimics, and Renilla luciferase vector pRL-CMV (Promega, Madison, WI) using the Lipofectamine 2000 transfection system (Invitrogen). After 24 hours of incubation, luciferase activity was analyzed by the Dual-Luciferase Reporter Assay System according to the manufacturer’s protocols (Promega).

### Animal surgery and tissue preparation

Thirty adult, male Sprague-Dawley (SD) rats were randomly divided into 5 groups according to different time points (6 rats each group). The animals were anaesthetized by an intraperitoneal injection of complex narcotics (85 mg/kg trichloroacetaldehyde monohydrate, 42 mg/kg magnesium sulfate, and 17 mg/kg sodium pentobarbital) before the sciatic nerve was exposed and lifted through an incision on the lateral aspect of the mid-thigh of the left hind limb. A 1 cm long segment of sciatic nerve was transected at the site proximal to its division of tibial and common peroneal nerves, and the incision site was then closed. To minimize discomfort and possible painful mechanical stimulation, animals were housed in large cages with sawdust bedding after surgery. Animals were allowed free access to water and food. At 0, 1, 4, 7, and 14 days after surgery, the animals in 5 groups were killed by decapitation, and the proximal sciatic nerve (0.5 cm long) was harvested for preparing the mRNA and protein samples. Animal experimentation was carried out in accordance with the NIH Guidelines for the care and use of laboratory animals (http://oacu.od.nih.gov/regs/index.htm) and ethically approved by the Administration Committee of Experimental Animals, Jiangsu Province, China (Approval ID: SYXK(SU)2007–0021).

### Real time reverse transcription polymerase chain reaction (RT-qPCR)

Total RNA was reversely transcribed to determine the expression of miR-sc8 (forward primer: 5' CGGTGAGGATTACGAAGAGGA 3' and reverse primer: 5' GTGCAGGGTCCGAGGT 3') using TaqMan® MicroRNA Reverse Transcription Kit (Applied Biosystems) and stem-loop RT primer (5' GTCGTATCCAGTGCAGGGTCCGAGGTATTCGCACTGGATACGACACCATC 3'). Total RNA samples were also reverse transcribed to cDNA using a prime-script reagent kit (TaKaRa, Dalian, China) to detect the expression of Egfr (forward primer: 5' CCCAACTAGGCACCTTTGAAG 3' and reverse primer: 5' GATGATCTGCAGGTTCTCCAAAG 3'). Quantitative real-time PCR was performed using SYBR Green Premix Ex Taq (TaKaRa) on an Applied Biosystems Stepone real-time PCR System. Amplification reaction protocol was performed for 40 cycles of 95°C for 10 min, 95°C for 15 s, and 60°C for 1 min. The relative expression was calculated using the comparative 2^−ΔΔCt^ method.

### Western blot analysis

Protein lysates were extracted from primary cultured cells or from proximal nerve tissues, and then lysed with cell lysis buffer (Biyuntian Biotechnology Co., Shanghai, China). The protein concentration was determined by the Micro BCA Protein Assay Kit (Pierce, Rockford, IL). The protein lysates were mixed with β-mercaptoethanol, glycerin, and bromophenol-blue for incubation at 95°C for 5 min. Equal amounts of protein samples were separated on 10% SDS-PAGE and electrotransferred to nitrocellulose membranes (Millipore, Bedford, MA). The membranes were blocked in 5% nonfat dry milk for 2 h, incubated with primary Egfr antibody (Abcam, Cambridge, MA) overnight at 4°C, and then incubated with HRP-conjugated secondary antibodies (Pierce). Membranes were developed with enhanced chemiluminescence reagent (Cell Signaling, Beverly, MA) and exposure to Kodak X-Omat Blue Film (NEN life science, Boston, MA). Measurements of band signal intensity were conducted with Grab-it 2.5 and Gelwork software.

### Statistical analysis

The data are presented as means±SD of 3 independent assays. Statistical analysis between groups was conducted by Student’s t-test with the software of SPSS 15.0 (SPSS, Chicago, IL), and statistical significance was set at *p* < 0.05.

## Results

### miR-sc8 affected SC proliferation and migration

After harvesting and purification, the obtained primary SCs were subjected to immunostaining for S100β (a specific SC marker) and Hoechst 33342 nuclear staining. The results showed that primary cultured SCs had a purity of more than 99% (data not shown). These highly pure SCs were used for all in vitro assays.

**Fig 1:**
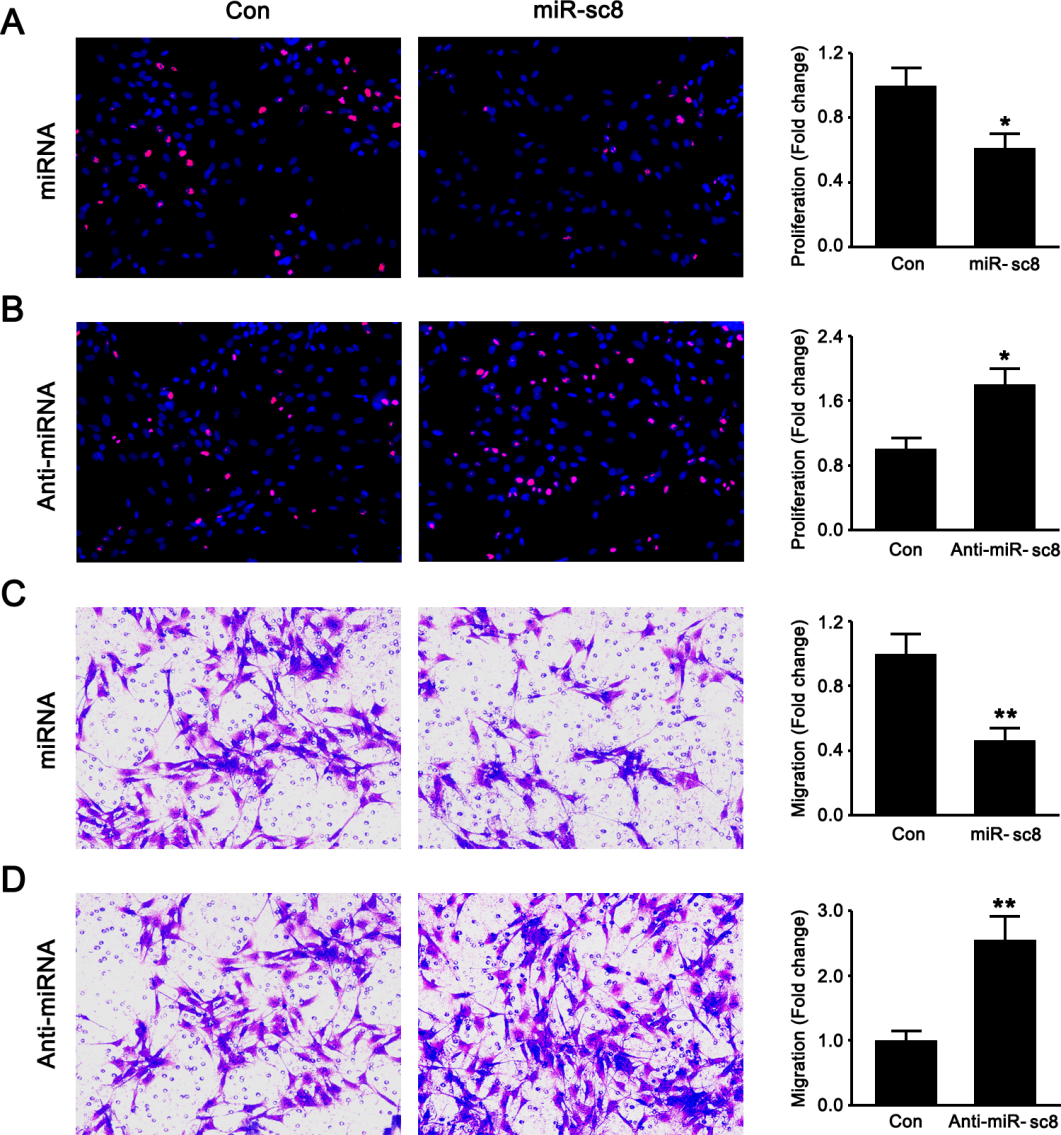
miR-sc8 inhibited SC proliferation and migration. After primary SCs were transfected with miR-sc8 mimic (miR-sc8), mimic control, miR-sc8 inhibitor (Anti-miR-sc8), or inhibitor control, representative images showed that SCs were double stained with EdU (red) plus Hoechst 33342 (blue) (**A, B**), and that SCs migrated to the bottom of the transwell chamber in a transwell-based migration assay (**C, D**). Histograms compared the cell proliferation and migration of primary SCs after the indicated treatments. Cell proliferation was calculated as the percentage of EdU-positive cells to cell population and normalized to control, while cell migration was also normalized to control. *P< 0.05 and **P< 0.01 versus control (Con).

To identify the effects of miR-sc8 on SC proliferation and migration, primary SCs were transfected with miR-sc8 mimic, miR-sc8 inhibitor, or non-targeting negative control. EdU-based proliferation assay was performed to determine whether miR-sc8 affected SC proliferation. Compared to transfection with non-targeting control, transfection with miR-sc8 mimic significantly decreased the proliferation rate of SCs to ~60% (Fig 1A), while transfection with miR-sc8 inhibitor dramatically increased the proliferation rate of SCs to nearly 2 folds compared to control (Fig 1B).

Transwell migration assay showed that transfection with miR-sc8 mimic reduced the migration ability of SCs to less than 50% when compared to that with non-targeting control (Fig 1C), while transfection with miR-sc8 inhibitor enhanced the migration ability of SCs compared to control (Fig 1D). These results suggested that miR-sc8 was able to inhibit the proliferation and migration of SCs.

### miR-sc8 directly targeted and negatively regulated Egfr

**Fig 2:**
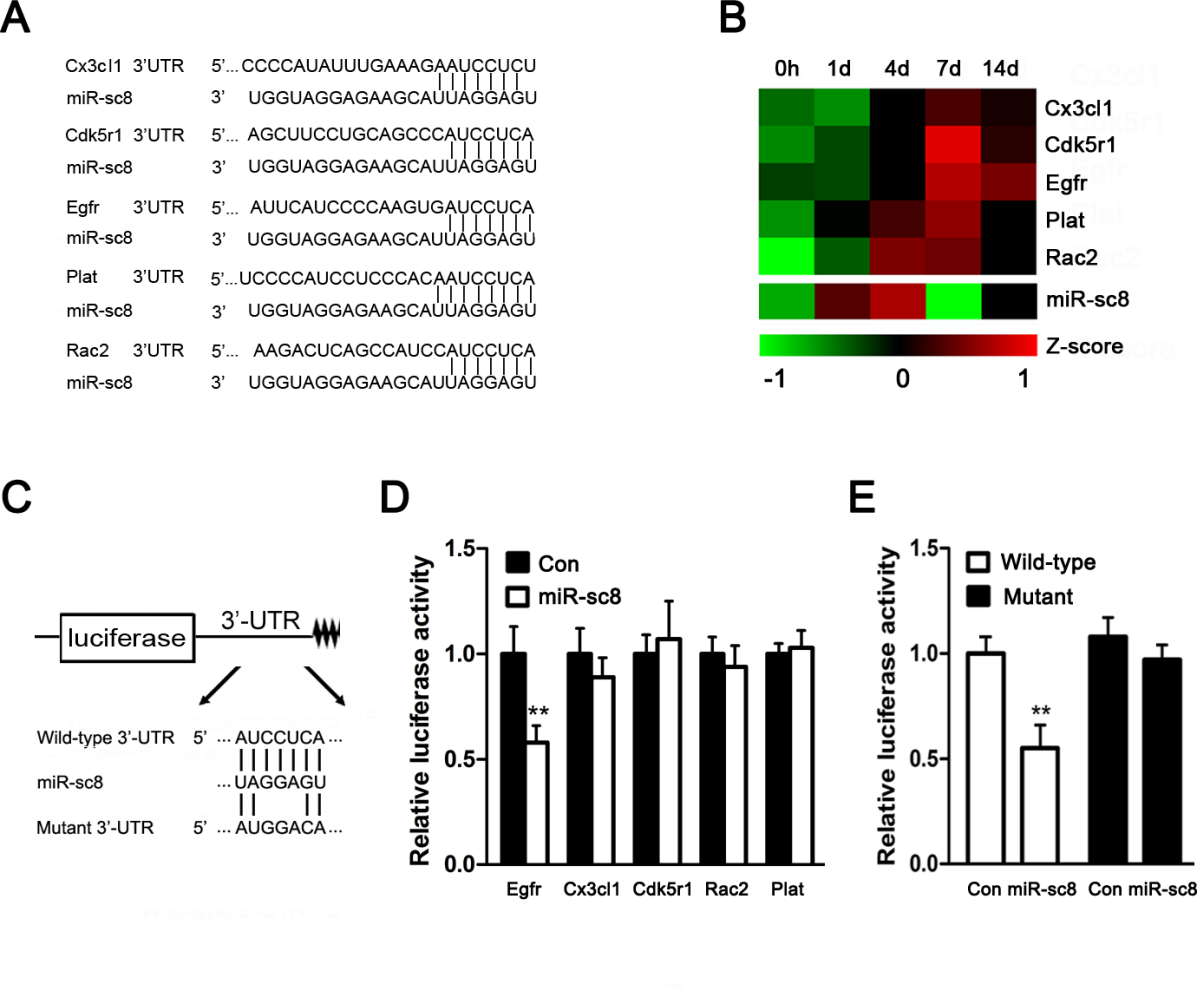
miR-sc8 directly targeted Egfr. (**A**) Predicted target sites of miR-sc8 at the 3’UTR of 5 potential targets genes for miR-sc8, including Cx3cl1, Cdk5r1, Egfr, Plat, and Rac2. (**B**) Heatmap of Cx3cl1, Cdk5r1, Egfr, Plat, Rac2 (from microarray) and miR-sc8 (from Solexa sequencing) at 0, 1, 4, 7, and 14 days in proximal nerve following sciatic nerve transection. (**C**) Schematic of wild-type and mutant p-Luc-UTR vector construction. (**D**) Histogram compared the relative luciferase activity after p-Luc-UTR vectors of Egfr, Cx3cl1, Cdk5r1, Rac2, or Plat were co-transfected into 293T cells with miR-sc8 mimic (miR-sc8) or mimic control (Con). (**E**) Histogram compared the relative luciferase activity after p-Luc-UTR vectors of the wild-type or mutant Egfr were co-transfected into 293T cells with miR-sc8 mimic (miR-sc8) or mimic control (Con). **P< 0.01 versus control (Con).

miRNAs perform their biological functions through directly binding to their target genes. The target prediction (Fig 2A), together with the negative regulation relationship as revealed by microarray data (Fig 2B) [14, 16], indicated that 5 genes, including Cx3Cl1, Cdk5r1, Egfr, Plat, and Rac2, might be potential targets of miR-sc8. To verify which one/ones of them were real target genes, the wild-type 3’-UTRs of these 5 genes were subcloned into the luciferase reporter vector, respectively (Fig 2C). We noted that miR-sc8 significantly suppressed the luciferase activity of the 3’-UTR of Egfr (Fig 2D), and mutation in the 3’-UTR of Egfr abolished the inhibitory effect of miR-sc8 on the relative luciferase activity (Fig 2E), suggesting that Egfr might be a direct target gene of miR-sc8.

### miR-sc8 regulated Egfr expression in SCs by translational suppression

**Fig 3:**
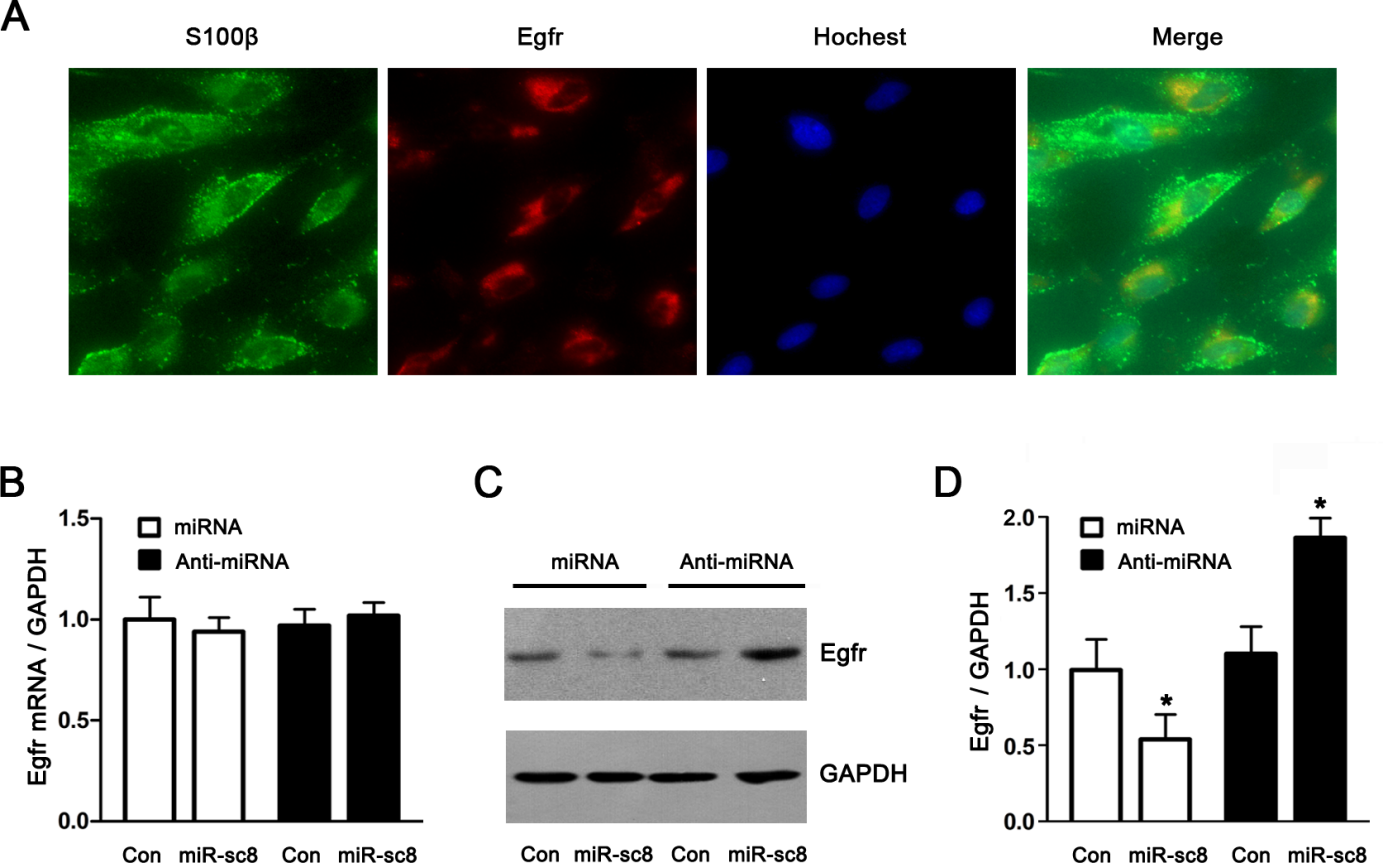
miR-sc8 regulated Egfr expression by translational suppression. (**A**) Immunostaining showed that Egfr was clearly expressed in primary SCs that had been labeled with S100β and subjected to Hochest 33342 nuclear staining. Also shown is the merge of 3 staining images. (**B**) Histogram compared the mRNA expression of Egfr in primary SCs transfected with miR-sc8 mimic (miR-sc8), miR-sc8 inhibitor (Anti-miR-sc8), or the corresponding controls. The mRNA expression of Egfr was normalized against GAPDH. (**C**) Representative Western blot images showed the protein expression of Egfr in primary SCs transfected with miR-sc8 mimic (miR-sc8), miR-sc8 inhibitor (anti-miR-sc8), or the corresponding controls. GAPDH served as a loading control. **(D)** Histogram compared the quantitative data of Western blot analysis as indicated in (C). *P< 0.05 versus the corresponding control (Con).

The expression of Egfr in primary SCs was determined, and Egfr was clearly expressed in primary SCs (Fig 3A). In general, miRNAs negatively regulate their target genes by mRNA degradation or translational repression. To verify the regulatory effects of miR-sc8 on the Egfr expression, primary SCs were transfected with miR-sc8 mimic, miR-sc8 inhibitor, and non-targeting negative control respectively, followed by the determination of the mRNA and protein expressions of Egfr. No significant differences in the mRNA expression of Egfr were observed in the cells after transfection with miR-sc8 mimic or inhibitor (Fig 3B). In contrast, the protein expression of Egfr was downregulated by overexpression of miR-sc8, or upregulated by silencing of miR-sc8 (Fig 3C and 3D). The results suggested that the regulation of the Egfr expression by miR-sc8 was roughly through translational repression rather than mRNA degradation.

### The miR-sc8 expression was negatively correlated with the Egfr expression in proximal nerve after sciatic nerve transection

**Fig 4:**
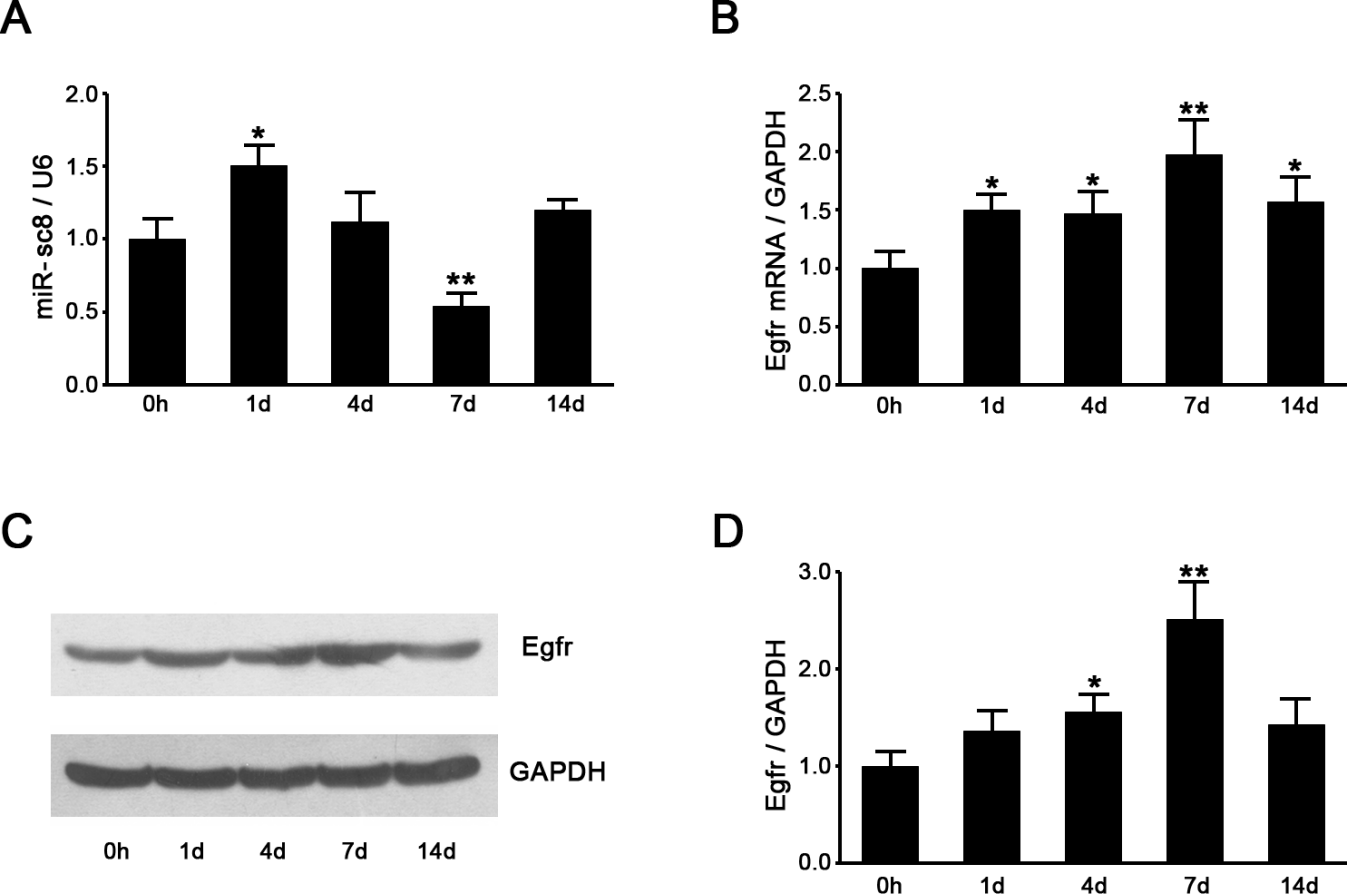
The expressions of miR-sc8 and Egfr were negatively intercorrelated following sciatic nerve injury. **(A)** Histogram compared the expression of miR-sc8 in proximal nerve at indicated different time points following nerve transection. The expression was normalized against U6. (**B**) Histogram compared the mRNA expression change of Egfr in proximal nerve segment at indicated different time points following nerve transection. The mRNA expression was normalized against GAPDH. (**C)** Representative Western blot images showed the protein expression of Egfr in proximal nerve segment at indicated different time points following nerve transection. (**D**) Histogram compared the protein expression change of Egfr in proximal nerve segment at indicated different time points following nerve transection. GAPDH served as a loading control. *P< 0.05, **P< 0.01 versus control (0 h following nerve transection).

The expression levels of miR-sc8 and Egfr in the proximal nerve following sciatic nerve injury were detected to evaluate the in vivo correlation between the expression of miR-sc8 and Egfr. RT-qPCR analysis showed that the miR-sc8 expression was increased at 1 day post nerve injury, and then significantly decreased at 7 days post nerve injury, followed by rebounding at 14 days post nerve injury (Fig 4A), which was consistent with Solexa sequencing data (Fig 2B). In contrast, the Egfr mRNA expression was constantly upregulated post nerve injury, and the Egfr mRNA expression at 7 days post nerve injury was increased to nearly 2 folds of that at 0 h post nerve injury (Fig 4B). The similar changes were observed for the Egfr protein expression, whose profile, however, was not exactly parallel to the profile of the Egfr mRNA expression (Fig 4C and 4D). If we compared the expression profile of miR-sc8 with that of Egfr, we would find that a negative correlation was present between the temporal expression profiles of miR-sc8 and Egfr. In other words, miR-sc8 could negatively regulate the expression of Egfr.

### Knockdown of Egfr recapitulated the effects of miR-sc8 on SCs

**Fig 5:**
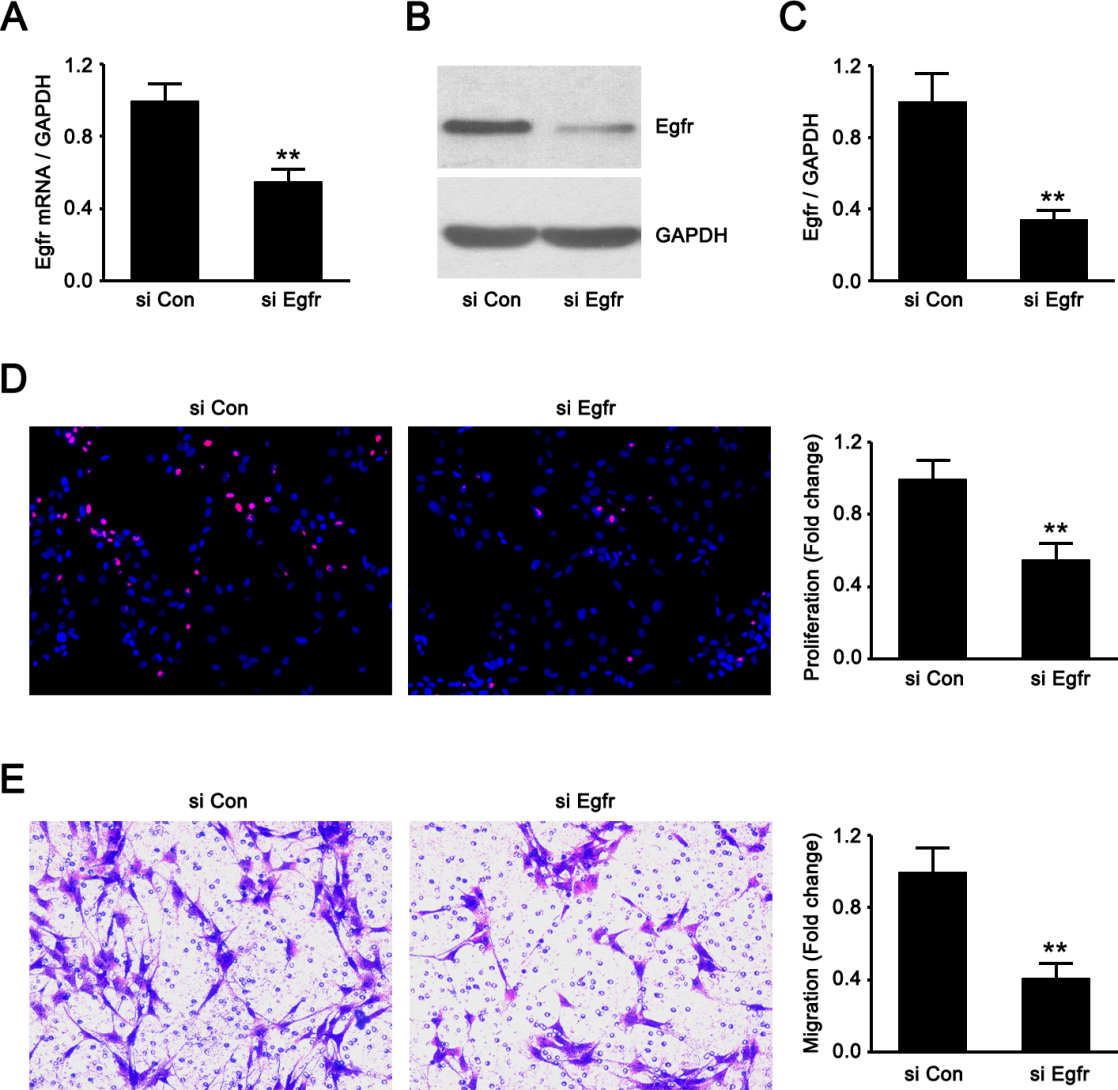
Egfr knockdown suppressed SC proliferation and migration. (**A-C**) Histograms compared the Egfr expression at mRNA and protein levels in primary SCs transfected with Egfr siRNA (si Egfr) and siRNA control (si Con), thus validating the efficacy of Egfr knockdown. Also shown **(B)** is representative Western blot images. GAPDH was used as an internal control. (**D**) Double staining with EdU (red) plus Hoechst 33342 (blue) showed that Egfr siRNA (si Egfr) inhibited SC proliferation compared to that of siRNA control (si Con). (**E**) Transwell-based migration assay showed that Egfr siRNA (si Egfr) inhibited SC migration compared to that of siRNA control (si Con). Also shown are the resulting histograms. **P< 0.01 versus siRNA control (si Con).

Egfr siRNA was synthesized and transfected into SCs. The determination of the Egfr expression at mRNA and protein levels confirmed the efficacy of Egfr knockdown (Fig 5A, 5B and 5C). Subsequently, we observed that Egfr knockdown significantly inhibited the proliferation and migration of SCs (Fig 5D and 5E), suggesting that downregulation of Egfr affected the phenotype modulation of SCs in the same manner as upregulation of miR-sc8 did.

### miR-sc8 affected proliferation and migration in SCs by targeting Egfr

**Fig 6:**
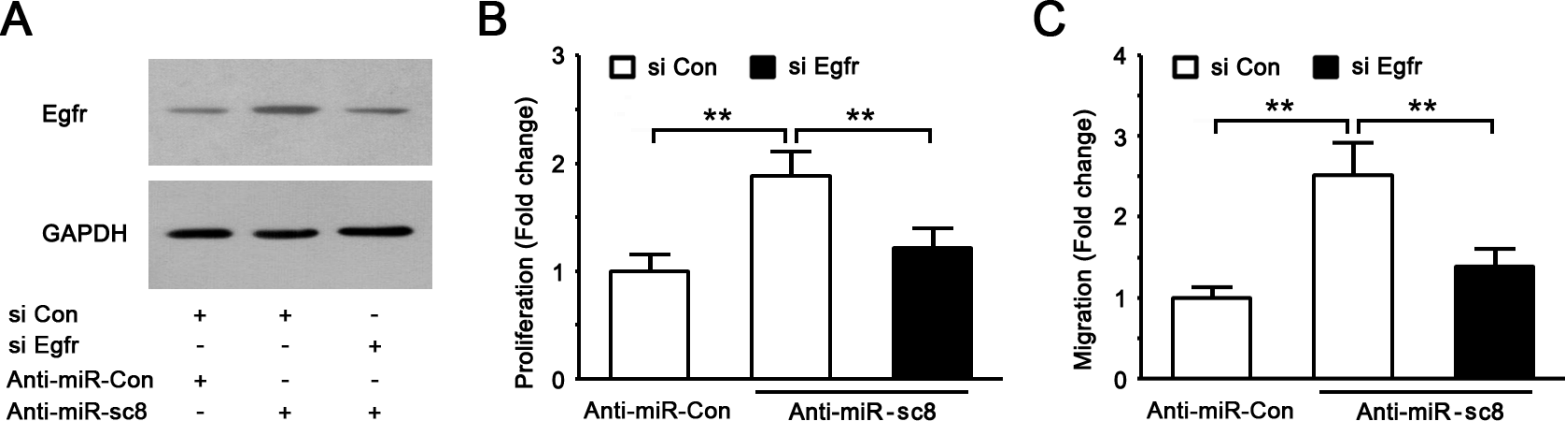
Egfr silencing reversed the promoting effect of miR-sc8 inhibitor on SC proliferation and migration. (**A**) Representative Western blot images showing the protein expression of Egfr in primary SCs that had been transfected with anti-control plus siRNA control, anti-miR-sc8 plus siRNA control, or anti-miR-sc8 plus Egfr siRNA. GAPDH served as a loading control. (**B**) Histogram compared cell proliferation of primary SCs after transfection with miR-sc8 inhibitors (Anti-miR-sc8) and with inhibitor control in the presence or absence of Egfr siRNA (si Egfr), respectively. (**C**) Histogram compared cell migration of primary SCs after transfection with miR-sc8 inhibitor (Anti-miR-sc8) and with inhibitor control in the presence or absence of Egfr siRNA (si Egfr), respectively. *P<0.05 versus inhibitor control (Anti-Con) and siRNA control (si Con).

Functional assays were conducted to test whether the inhibitory effects of miR-sc8 on SC proliferation and migration was directly through suppression of the Egfr expression. To address this issue, primary SCs were transfected with miR-sc8 inhibitor in the presence or absence of Egfr siRNA. The Egfr expression in the cells transfected with miR-sc8 inhibitor plus siRNA control was increased compared to that transfected with inhibitor control plus siRNA control, and the Egfr expression in the cells transfected with Egfr siRNA plus miR-sc8 inhibitor was similar to that transfected with inhibitor control plus siRNA control (Fig 6A), suggesting that Egfr siRNA might attenuate miR-sc8 inhibitor-induced increase in the Egfr expression to a certain degree. More importantly, the proliferation rate or migration ability of primary SCs transfected with miR-sc8 inhibitor were remarkably increased compared to that transfected with siRNA control plus inhibitor control, respectively (Fig 6B and 6C). Co-transfection of SCs with miR-sc8 inhibitor and Egfr siRNA, however, significantly attenuated the miR-sc8 inhibitor-induced increase in the proliferation rate or the migration ability of SCs respectively (Fig 6B and 6C).

## Discussion

During peripheral nerve injury and regeneration, the complex molecular and cellular interactions are precisely orchestrated to result in a series of morphological and functional changes. Among these changes, SCs proliferate and migrate to form bands of Bungner, and help to myelinate the regrowing axons [17, 18], thus contributing to the creation of a favorable microenvironment for nerve regeneration [19]. Obviously, a better understanding of the cellular and molecular mechanisms underlying peripheral nerve injury will benefit the development of peripheral nerve repair strategies.

We have previously used Solexa sequencing to identify a group of novel miRNAs, which are differentially expressed in the injured nerve following sciatic nerve transection [14, 15]. Since miRNAs play regulatory roles during peripheral nerve regeneration, the goal of this study was set at determining the biological functions of miR-sc8, a member of the above identified miRNA group.

Owing to the involvement of SC phenotype modulation in peripheral nerve regeneration, the influence of novel miRNAs on proliferation and migration of SCs definitely attracts the research interest. At first, in this study, we observed that miR-sc8 inhibited SC proliferation and migration. Then, in order to determine the action mechanisms of miR-sc8, target prediction algorithm and dual-luciferase reporter assay were performed to confirm that Egfr was a real target gene of miR-sc8. Furthermore, we observed that miR-sc8 suppressed Egfr expression possibly through inhibiting Egfr translation rather than causing Egfr mRNA degradation. Egfr, also known as ErbB1, is a member of the ErbB family of receptors. Upon ligand binding, Egfr elicits several downstream signaling cascades, principally the MAPK, Akt, and JNK signaling pathways, and leads to DNA synthesis, cell proliferation, cell migration, and adhesion. The overexpression and/or overactivity of Egfr are associated with the development of many cancers, and thus some anticancer therapies are designed against Egfr [20–23]. There have been numerous publications reporting that different miRNAs regulate cancer development by targeting Egfr. For example, miR-7 inhibits the invasion and metastasis of cancer cells by regulating Egfr expression [24–26]; miR-145 inhibits cell proliferation of lung adenocarcinoma by targeting Egfr [27], miR-146a suppresses tumor growth and progression by targeting Egfr in prostate cancer [28]; miR-27a regulates non-small lung cancer by targeting Egfr [29]. Our current results demonstrated that Egfr, as an oncogene, also participated in regulation of peripheral nerve regeneration.

To have a deep insight into an in vivo regulation of Egfr by miR-sc8, we noted that the temporal change of Egfr protein expression in the injured nerve was not completely consistent with that of its mRNA expression in the injured nerve. This incomplete matching was observed from the different expression change from 0 h to 1 day post nerve injury between the Egfr mRNA and its protein. Meanwhile, the expression of miR-sc8 was found to be significantly increased during the same time period post nerve injury, which might account for no significant increase of the protein expression of Egfr from 0 h to 1 day post nerve injury. The results suggested that an in vivo negative regulation of Egfr by miR-sc8 at the posttranscriptional level might be through translational suppression.

Primary SCs were transfected with Egfr siRNA, and the resulting downregulation of Egfr inhibited proliferation and migration of SCs. On the other hand, Egfr knockdown attenuated the miR-sc8 inhibitor-induced increase in proliferation and migration of SCs. The observation that Egfr knockdown recapitulated the inhibitory effects of miR-sc8 on SC proliferation and migration contributes to the identification of Egfr as a functional mediator of miR-sc8.

In conclusion, peripheral nerve injury may lead to downregulation of the miR-sc8 expression, which in turn upregulates the Egfr expression, thus promoting proliferation and migration of SCs. Overall, our data reveal that miR-sc8 affects phenotype modulation of SCs by targeting Egfr, providing further insights into the regulatory role of miRNAs in peripheral nerve regeneration.
